# The role of astrocytes in oxidative stress of central nervous system: A mixed blessing

**DOI:** 10.1111/cpr.12781

**Published:** 2020-02-08

**Authors:** Yaxing Chen, Chen Qin, Jianhan Huang, Xin Tang, Chang Liu, Keru Huang, Jianguo Xu, Gang Guo, Aiping Tong, Liangxue Zhou

**Affiliations:** ^1^ Department of Neurosurgery West China Medical School West China Hospital Sichuan University Chengdu China; ^2^ State Key Laboratory of Biotherapy West China Medical School Sichuan University Chengdu China

**Keywords:** astrocyte, astrogliosis, central nervous system, oxidative stress, RNS, ROS

## Abstract

Central nervous system (CNS) maintains a high level of metabolism, which leads to the generation of large amounts of free radicals, and it is also one of the most vulnerable organs to oxidative stress. Emerging evidences have shown that, as the key homeostatic cells in CNS, astrocytes are deeply involved in multiple aspects of CNS function including oxidative stress regulation. Besides, the redox level in CNS can in turn affect astrocytes in morphology and function. The complex and multiple roles of astrocytes indicate that their correct performance is crucial for the normal functioning of the CNS, and its dysfunction may result in the occurrence and progression of various neurological disorders. To date, the influence of astrocytes in CNS oxidative stress is rarely reviewed. Therefore, in this review we sum up the roles of astrocytes in redox regulation and the corresponding mechanisms under both normal and different pathological conditions.

## 1 INTRODUCTION

Central nervous system maintains a high metabolic rate, accounting for 20% of the overall energy consumption but only 2% of body mass.^123^ Such high energy consumption yields large amounts of free radicals, such as reactive oxygen species (ROS) and reactive nitrogen species (RNS). Oxidative stress occurs when the production of free radicals exceeds the antioxidant capacity of CNS. Modern molecular pathophysiology studies have confirmed that oxidative stress plays an important part in various pathological changes in CNS, such as hypoxic/toxic injury, metabolic disturbance, inflammation and oncogenesis.^1^, ^28^, ^60^, ^110^, ^127^ Specifically, ROS and RNS could be overproduced under various CNS pathogenesis such as abnormal cell metabolism, mitochondrial damage and calcium overload, which disturbed the balance between physical oxidative reaction and antioxidative system and thus generated lipid, protein peroxidation or DNA damage on the neurons which lead to the damage of neurons.^48^ Previously, ROS were considered more as a detrimental substance which might cause cell damage and lead to various pathological process in CNS. Along with the in‐depth study of redox biology, ROS/RNS is also regarded as an important signal molecule which regulates various CNS activities.^100^

**Figure 1:**
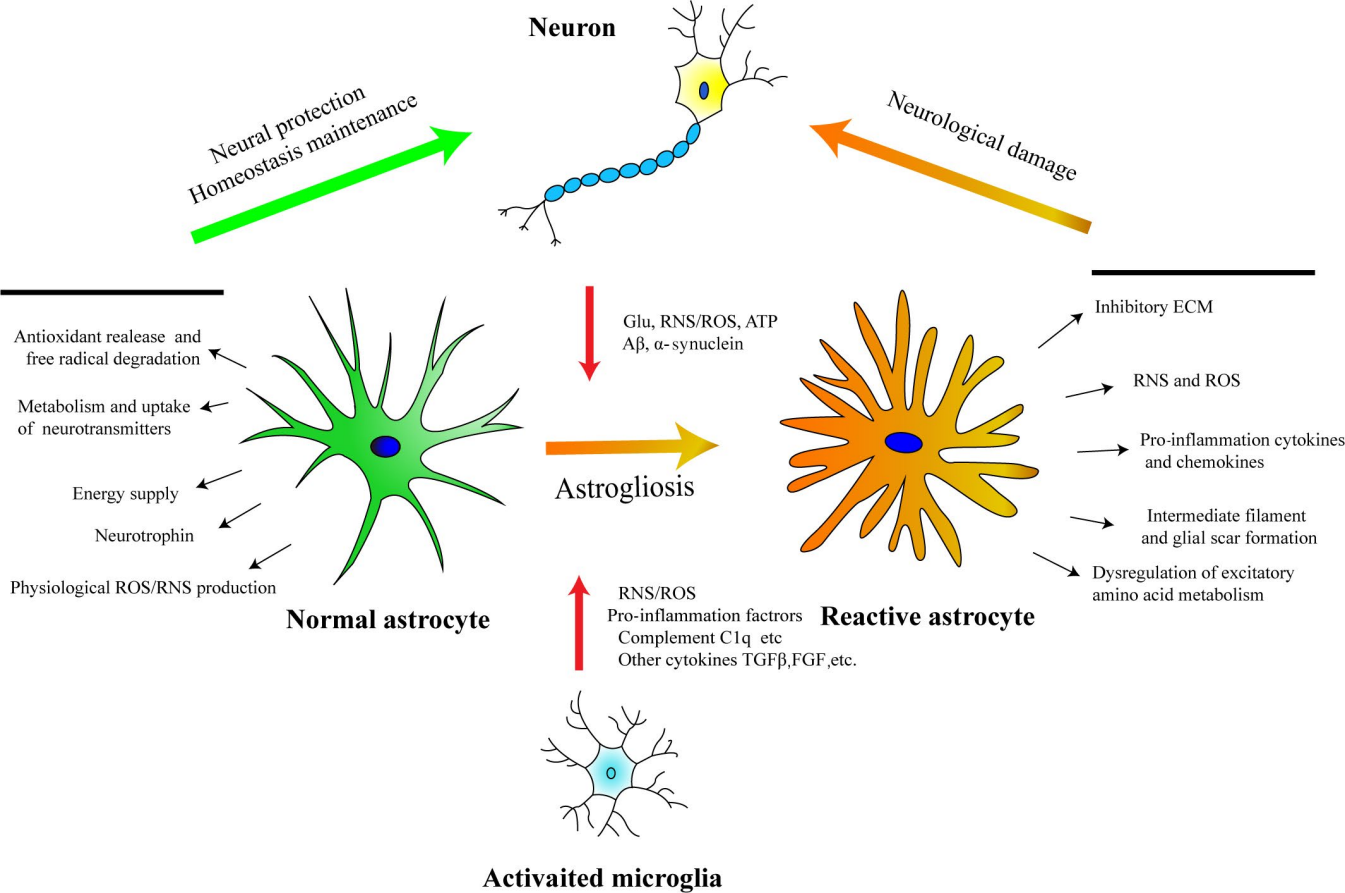
The main molecular basis of response and the interaction among astrocyte, microglia and neuron. Under physiological status, astrocytes maintain homeostasis by releasing antioxidants, degrading ROS/RNS, providing energy and neurotrophin, uptake and metabolism of neurotransmitters, etc Under pathological conditions, astrocytes could be activated via stimulation from activated microglia and degenerated neurons, causing excessive secretion of free radicals and pro‐inflammatory cytokines, glial scar formation and inhibitory ECM deposition, dysregulation of excitatory amino, etc, which lead to aggravation of neurological damage^96^

Astrocytes are the key homeostatic cells in the CNS, playing a crucial role in maintaining physiological CNS function such as providing nutrition to neurons, keeping the integrity of blood brain barrier, regulating synapse activity and processing cell metabolites.^130^ As the research progressed, increasing evidence has revealed the crucial role of astrocytes in regulating oxidative stress in CNS. On the one hand, a complete antioxidant response in astrocytes promotes the decomposition and clearance of free radicals produced by neurons and other cell types in the CNS thus protecting the central nervous system from oxidative stress damage. On the other hand, under certain pathological conditions, astrocytes may act as one of the main sources of detrimental ROS and RNS and these excessive free radicals can promote the activation of microglia or directly cause neural damage^15^, ^30^, ^117^, ^118^, ^125^ (Figure 1). As the main inherent immune cell of central nervous system, oxidative stress in CNS is well studied in microglia, while there are few reviews to summarize the role of oxidative stress in the CNS in the perspective of astrocytes. This review aims at summarizing the reported role of astrocytes in CNS oxidative stress regulation and the effects of oxidative stress on the physiological or pathological functions of astrocytes to provide a new direction for future intervention in CNS diseases.

## 2 OXIDATIVE STRESS AND ANTIOXIDANT SYSTEM OF THE CENTRAL NERVOUS SYSTEM

### 2.1 The oxidative system of the central nervous system

In the CNS, there are two main sources of endogenous ROS: mitochondria and NADPH‐oxidized (NOX) pathway.^48^ Mitochondrial ROS (mROS) is mainly produced via the process of electron transfer which account for most of the total ROS.^76^ For another important source of ROS, seven NOXs (NOX1‐5, DUOX1, DUOX2) have been found specifically expressed on the cell membrane of different cell types, catalysing the transport process of electrons from NADPH to O_2_ which eventually convert to ROS.^9^, ^90^ Apart from those two main sources, ROS can also be produced by other oxidase such as cytochrome P450, xanthine oxidase, lipoxygenase and myeloperoxidase.^90^ RNS is mainly produced from a kind of amino acid, L‐arginine, via the metabolic process of arginine in mitochondria, which is mainly catalysed by nitric oxide synthase (NOS).^24^

### 2.2 The antioxidant system of CNS

Two major types of antioxidant response are exist in the CNS: the non‐enzymatic antioxidant system and the enzymatic antioxidant system.^69^, ^147^ The enzyme antioxidant system mainly includes several antioxidant enzymes such as glutathione peroxidase (GPxs), superoxide dismutase (SODs), catalase (CATs) and peroxidase (Prxs), and some phase II reaction enzymes, such as haeme oxygenase 1 (HO‐1), reduced coenzyme/quinone oxidoreductase 1 (NQO1) and γ‐glutamylcysteine acid ligase (GCLC).^147^ Nuclear factor erythroid 2‐related factor 2 (Nrf2) is an important transcription factor involved in maintenance of redox and metabolic homeostasis by regulating the expression of various antioxidant enzymes.^22^ Non‐enzymatic antioxidant systems include a variety of endogenous reducing substances, such as vitamin C, vitamin E, glutathione (GSH), NADPH, uric acid, bilirubin and melatonin, playing crucial role in ROS scavenging.^13^, ^147^ Besides, the thioredoxin (Trx) system is another non‐enzymatic antioxidant system in the CNS^89^, ^100^ and it acts as an antioxidant through the reversible oxidation‐reduction reaction between the active site of cysteine in Trx and NADPH.^82^

## 3 OXIDATIVE STRESS GENERATED BY ASTROCYTES

### 3.1 Mitochondria‐derived oxidative stress in astrocytes

Mitochondrial metabolism plays a crucial role in astrocytic redox regulation under physiological or pathological conditions.^49^ According to the previous view, mitochondria are only distributed in the cell body of astrocytes. However, emerging evidences have confirmed that mitochondria are also present in its thin and long processes, indicating a more complicate function of astrocytic mitochondria.^115^ Abnormal structure and function of astrocytes have been reported to be involved in clinical pathogenesis of Alexander's disease and amyotrophic lateral sclerosis (ALS).^55^, ^73^ In a study focused on experimental ALS, mitochondrial dysfunction is found in SOD1^G93A^‐expressing astrocytes which greatly contribute to motor neuron damage in the spinal cord and that damage of motor neurons could be attenuate by some mitochondrial‐specific antioxidants.^16^ To study the role of astrocytic electron transport chain (ETC) in neurological disorders, an astrocytic mitochondrial transcription factor A (Tfam) conditionally knockout model was established which showed increased neuronal death induced by photochemically initiated thrombosis‐induced ischaemic stroke.^32^ Mice with astrocyte‐specific deletion of the mitochondrial m‐AAA protease (an important protease maintaining mitochondrial homeostasis by degrading misfolded polypeptides and proteins) shows neuronal impairment and behaviour defect.^87^ Previous studies have suggested that the level of insulin‐like growth factor‐1 (IGF‐1) is closely associated with neuronal ageing and neurodegeneration diseases, and a recent study reveals that IGF‐1 singling could modulate the function of mitochondria and redox level in astrocytes.^70^ Another study confirms that mROS mediate the classical NLRP3 inflammasome activation induced by LPS in astrocytes.^5^ Methamphetamine (METH), a monoaminergic toxin, causes death of dopamine terminals and leads to astrogliosis in vivo by disrupting mitochondrial function and increasing ROS in astrocytes.^62^

### 3.2 NADPH‐derived oxidative stress in astrocytes

The family of NOXs contains 7 members, among which NOX2 and NOX4 are considered as the most abundant NOXs isoforms expressed in the CNS.^120^ Although it has been reported that NOX2 is mainly expressed in microglia^40^ and NOX4 is only expressed slightly in astrocytes,^39^ recent studies have shown that even the low expressed NOX in astrocytes also plays an important role in the regulation of oxidative stress in the central nervous system. NOX activity and superoxide level of astrocytes increase with ageing,^11^ and they are closely related with various diseases. In an Alzheimer's disease model, amyloid‐β could upregulate astrocytic NOX2 which induced astrogliosis.^17^ In the lipopolysaccharide (LPS)‐induced PD model, the expression of NADPH oxidase complex is increased and it is significantly involved in the pathogenesis of PD.^114^ HIV‐1 glycoprotein 120 (gp120) is a well‐known capsid protein of human immunodeficiency virus which is closely related to the AIDS‐induced neurotoxicity. Results from an in vitro study demonstrate that gp120 and methamphetamine (MA) may cause apoptotic cell death by inducing oxidative stress through NADPH oxidase (NOX) and other pathways in primary and SVGA astrocytes and such effects could be further confirmed by inhibiting NADPH‐derived ROS generation using NOX specific inhibitor or siRNA.^113^ In an in vitro hypoosmotic swelling model, the amount of ROS produced by NADPH oxidase is found to increase significantly in astrocytes.^99^ On the contrary, oxidative stress is known to cause astrocyte swelling which contributes largely to the whole brain oedema.^52^ To sum up, NADPH oxidase significantly affects the physiological function of astrocytes and more attention should be paid to astrocytic NADPH oxidative stress when it comes to seeking new methods to modulate NOX activity in the CNS.

### 3.3 RNS produced in astrocytes

Astrocytic RNS production is another important part of the astrocyte‐derived oxidative stress. There are three main NOS isoforms expressed in the CNS, namely the Ca2+/calmodulin‐dependent neuronal NOS, the endothelial NOS and the Ca2+‐independent inducible NOS (iNOS), and evidence suggests that all three NOS isoforms are expressed in astrocytes.^35^, ^36^, ^85^ An in vitro study confirms that LPS stimulation can increase the NO production in astrocytes.^84^ In a primary astrocyte‐neuronal co‐culture system, cytokine stimulation could increase astrocytic RNS production causing the dysfunction of complexes II, III and IV in neurons and this effect could be reversed when astrocytes are removed.^121^ Severe systemic inflammation has been reported to cause brain injury via activating astrocytic iNOS, nuclear factor kappa B (NF‐κB) and some other pathways.^10^ Alexander diseases have been proved to be closely associated with glial dysfunction, and a recent study confirmed that astrocytic NO is significantly involved in the astrocyte‐induced neuronal degeneration by affecting cGMP signalling in neurons.^135^ Additionally, emerging evidences have revealed the importance of S‐nitrosylation (S‐nitrosylate cysteine thiols in target proteins) in astrocytic NO signalling. For example, NO‐induced S‐nitrosylation of PDI (protein disulphide isomerase) is enhanced in an in vitro ischaemic/reperfusion model, causing SOD1 aggregation in astrocytes which might be involved in the pathogenesis of CNS ischaemic/reperfusion injury.^18^

## 4 THE ANTIOXIDANT RESPONSE OF ASTROCYTES

### 4.1 Excitatory amino acids regulated by astrocyte

**Figure 2:**
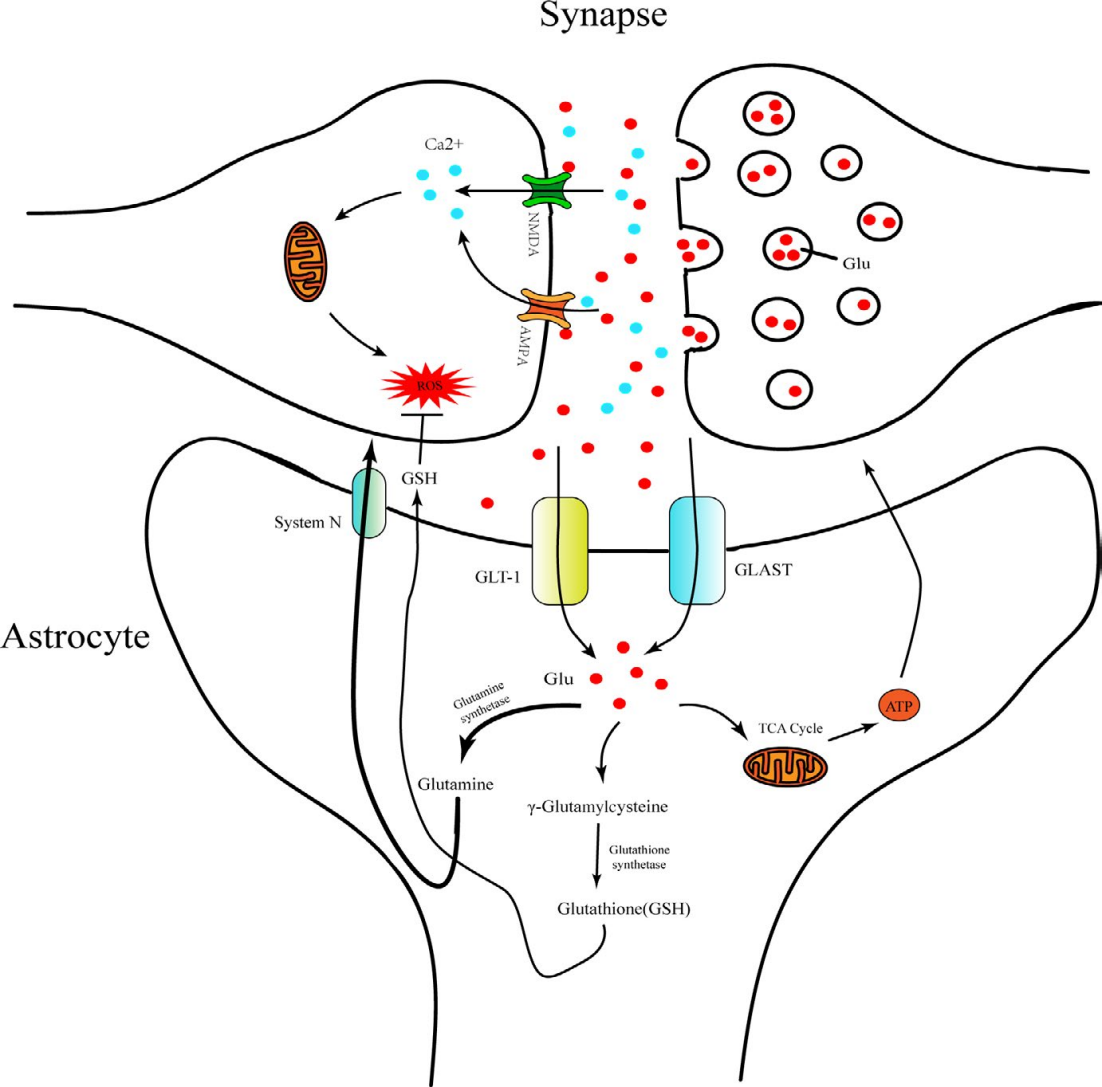
The role of astrocytes in oxidative stress regulation associated with glutamate uptake and metabolism. Under pathological conditions, excessive excitatory neurotransmitters such as glutamate were released from pre‐synaptic membrane and accumulated in the synaptic cleft which activating specific glutamate receptor NMDA and AMPA. Such activated glutamate receptor allows a large influx of Ca2 + which further leads to mitochondrial calcium overload and ROS generation. In another aspect, glutamate in synaptic cleft could be transported into astrocyte by some specific glutamate transporters like GLT1 and GLAST which are highly expressed on the cell membrane of astrocytes adjacent to the synapse. Most of glutamate accumulated in astrocytes is converted into glutamine and be delivered to neurons by the glutamine transporter (system N) maintaining excitatory neurotransmission.^126^ Partial glutamate in astrocytes is converted into ATP via TCA cycle in the mitochondria and GSH, respectively, catalysed by glutathione synthetase in cytoplasm. These glutamate metabolites returned into the intercellular space providing energy to neurons and inhibiting ROS/RNS^130^

Glutamate is an important excitatory neurotransmitter mainly released by excitatory neurons delivering excitatory signal in CNS. However, excessive glutamate in synaptic cleft may lead to calcium overload by overactivating NMDA receptors or non‐NMDA receptors (including AMPA receptors and kainic acid (KA) receptors) which generate large amounts of ROS and lead to neurotoxicity.^107^ Astrocytes are the main cells to maintain homeostasis of glutamate which indirectly affect the balance of oxidative stress (Figure 2). For example, in a study focused on the function of glutamate transporter in CNS, three glutamate transporters (GLAST, GLT‐1 and EAAC1) were, respectively, knocked down in vivo and in vitro and the results indicate that elevated extracellular glutamate and excitotoxicity mainly appeared in the astrocytic glutamate transporters' (GLAST and GLT1) knockdown group compared with EAAC1 (neuronal glutamate transporter) knockdown group, which suggested the central role of astrocytes in functional glutamate transport and prevention of glutamate neurotoxicity.^105^ In a transgenic rat model of SOD1 mutant‐mediated amyotrophic lateral sclerosis (ALS), researchers found significantly loss of GLT1 (EAAT2) in the ventral horn of the spinal cord and this change appeared before the motor neuron damage, suggesting a potential role for GLT1 dysfunction and oxidative glutamate toxicity in ALS pathology.^46^ In another study, a mouse model with a mutant caspase‐3 consensus site in the EAAT2 sequence was generated which shows delayed disease progress and longer lifespan compared with the wild‐type mice when crossed with SOD1‐G93A ALS mice.^104^ Apart from ALS, some neurotoxic substances such as titanium dioxide (TiO_2_)^138^ and methylmercury^68^, ^143^ or the pathological conditions such as oxygen/glucose deprivation^23^, ^38^ and could lead to abnormal glutamate transport which further result in oxidative stress and neurotoxicity.

### 4.2 GSH synthesis in astrocytes

Compared with neurons, astrocytes have higher capacity for the GSH production and storage, and they can protect neurons from oxidative damage by releasing GSH into the extracellular microenvironment.^8^, ^19^, ^44^ De novo synthesis of GSH in the brain mainly relies on Sxc^−^ cystine/glutamate antiporter (also known as Sxc^−^_,_ mainly expressed on the membrane of astrocytes and very little in neurons) which exports glutamate from the cells in exchange for cystine providing the raw material for glutathione synthesis,^130^ and then, the accumulated cystine in astrocytes can be converted into glutathione catalysed by γ‐glutamate‐cysteine ligase and GSH synthetase.^77^ A study has confirmed that enhancing Sxc‐ expression in astrocytes could increase the GSH level and providing neuroprotection effect.^112^ Except for the direct provision of GSH, astrocytes also supply glutathione precursor, CysGly, for neuronal glutathione generation which catalysed by astroglial ectoenzyme γ‐glutamyl transpeptidase.^26^ Additionally, astrocytic GSH synthesis has been reported to be regulated by some inflammation‐related signal pathways. For example, after conditional knockout of astrocyte STAT3, the GSH content of astrocytes is significantly lower than that the wild‐type cells accompanying with the lower mitochondrial membrane potential and the higher level of ROS.^109^ An in vitro study shows that IL‐1β can increase the production of GSH in astrocytes by activating NF‐KB.^43^ Some antioxidant drugs such as pramipexole, nitrogen acetylcysteine (NAC) and zonisamide have been found helpful for the treatment of Alzheimer's disease, Parkinson's disease and many other degenerative diseases of the central nervous system by increasing GSH synthesis in astrocytes.^33^

### 4.3 Nrf2‐keap1‐ARE antioxidative pathway of astrocyte

**Figure 3:**
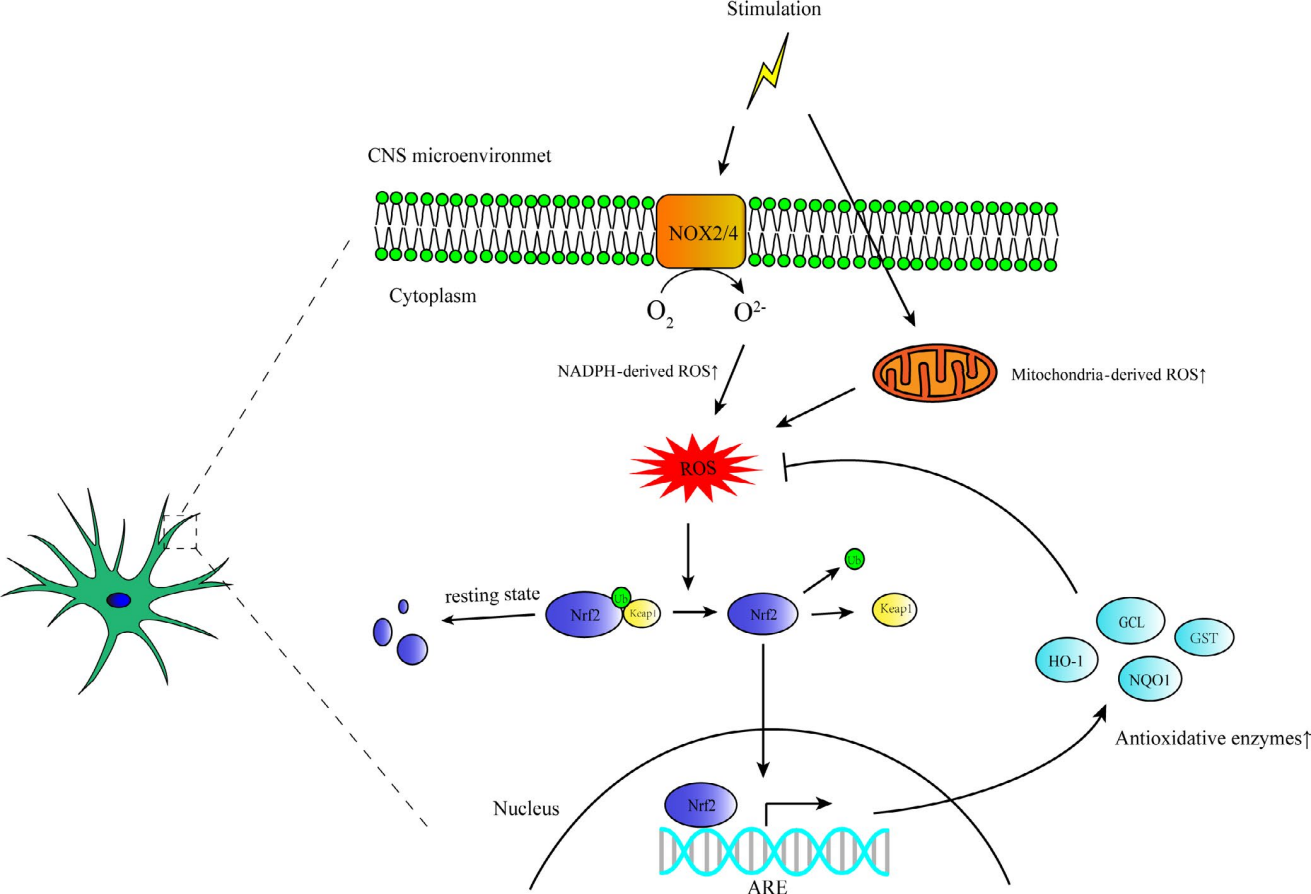
The relationship of oxidative stress and Nrf2‐regulated antioxidant response in astrocytes. Pathological stimulus in CNS microenvironment causes NOX activation or mitochondrial dysfunction both leading to excessive ROS generation in astrocytes which directly interact with keap1 causing the decrease of the activity of ubiquitin E3 ligase and thus prevents the degradation of Nrf2. The stabilized Nrf2 enters the nucleus and binds to ARE promoting the transcription of phase II antioxidative enzymes such as HO‐1, NQO‐1, GCL and GST, thus inhibiting astrocyte‐derived oxidative stress^146^

Nrf2‐keap1‐ARE pathway is an important endogenous antioxidant system in CNS. In this antioxidative system, Nrf2 is an inducible transcription factor which can be activated in response to oxidative stress^146^ (the specific signal pathway for Nrf2 activation is shown in Figure 3). It is reported that a specific activator of Nrf2, tBHQ, could enhance the activation the Nrf2 and the downstream antioxidative enzymes like NQO1 and GSTP1 in astrocytes while weakly in neurons.^2^ In another study, a ARE reporter was transiently transfected into the brain slice and primary cultures and the results indicating that high level of ARE activation and the downstream antioxidative gene expression is mainly restricted to astrocyte cell populations.^86^ Some researchers found that endogenous H_2_O_2_ generated in astrocytes under certain conditions could protect neurons from oxidative stress by activating Nrf2 and further activate the antioxidant stress response.^41^ Hyperbaric oxygen preconditioning can enhance the expression of Nrf2 in astrocytes but not neurons which increases the tolerance to ischaemic injury of spinal cord.^139^ In a Parkinson's model, tertiary butylhydroquinone (tBHQ) can activate astrocytes' Nrf2 to protect against neurotoxicity induced by MPP^+^ 4. Conditional knockout of keap1 in astrocytes could significantly alleviate the demyelinating damage in a mouse model of multiple sclerosis by activating Nrf2.^25^ In an in vitro study, primary Nrf2^−/−^ astrocytes show more severe inflammatory response and cell damage effect than the wild‐type astrocytes do under a pro‐inflammatory stimulus^93^ All these evidences suggest that astrocytic Nrf2 is the main regulator involved in CNS oxidative homeostasis and it might be a promising target for neuroprotection.

## 5 THE EFFECT OF MICROENVIRONMENTAL OXIDATIVE STRESS ON ASTROCYTES

### 5.1 Mediating the inflammatory response of astrocytes

Studies suggested that astrocytes are widely involved in inflammation response and innate immunity of the central nervous system. Reactive astrogliosis, also known as astrogliosis, is a general pathologic change in many CNS disorders. Concretely, overactive astrocyte persistently secretes large amounts of inflammatory factors and aggravate neuronal damage.^66^, ^117^ Oxidative stress plays an important role in astrocyte‐associated inflammation and the process of astrogliosis.^102^, ^117^ Free radicals can activate a variety of inflammatory‐related signalling pathways in astrocytes and promote the inflammatory factor release.^53^ NLRP3 inflammasome is a kind of intracellular ROS‐activated protein complexes that plays an important role in the innate immune response.^133^ Studies have shown that mitochondria‐derived ROS can activate the astrocytic NLRP3 inflammatory cascade by promoting the cleavage of pro‐caspase‐1 and the cleaved caspase‐1 could further cleave IL‐1β and IL‐18 precursors to promote the release of IL‐1β and IL‐18.^122^, ^148^ Another study confirmed that uncoupling protein 2 (UCP2), a member of mitochondrial anion carrier proteins (MACP), can inhibit astrocytic maturation of IL‐1β by reducing mitochondrial ROS in astrocyte.^27^ Additionally, chronic ethanol stimulation was found to activate NLRP3‐related inflammation in astrocyte by increasing mitochondria‐derived ROS.^5^ Similarly, NADPH oxidative‐derived ROS was also been found to be participant in the inflammatory response induced by astrocytes under the treatment of LPS or IFN‐γ.^94^ Except for the astrocyte‐derived oxidative stress, antioxidant response of astrocytes is also regulating the inflammatory response of CNS. For instance, astrocytes were reported to help increasing the expression of HO‐1 in microglia, decreasing the production of microglia‐derived ROS, thereby inhibiting excessive inflammation of CNS.^80^ Nrf2‐Keap1‐ARE, a main endogenous antioxidant stress signalling pathway, is also known to be an important anti‐inflammation pathway. In a study focused on astrocytes, researchers found that NF‐KB singling is more likely to be activated in the Nrf2 knockout cells than the wild‐type cells do and lead to more pro‐inflammatory factors released in an mechanical scratch model.^93^ Furthermore, a variety of other inflammatory signalling pathways have been found to be inhibited by activating Nrf2 signal pathway in astrocytes.^54^, ^141^

### 5.2 Reactive astrogliosis and glial scar formation

Reactive astrogliosis involved in a wide range of CNS disorders such as neurotrauma, stroke, perinatal asphyxia, brain haemorrhage, CNS infections, epilepsy or AD,^96^ mainly characterized by accelerated proliferation, cell hypertrophy and migration under various stimulation. On the molecular scale, such activation is associated with high expression level of certain cytoskeletal proteins such as glial fibrillary acidic protein (GFAP), vimentin (Vim) and increased secretion of extracellular matrix such as chondroitin sulphate proteoglycan (CSPG).^96^ In the later stage of CNS injury, astrogliosis may lead to glial scar formation which is considered as the main physical barrier that inhibits the axon regeneration of neurons. So the formation of glial scar is widely considered as one of the important factors affecting the recovery of neural function in spinal cord injury (SCI) or traumatic brain injury (TBI).^34^, ^57^, ^144^ A growing evidence suggests that oxidative stress and inflammation are significant factors in promoting astrogliosis and glial scar formation^117^ (specific factors that affect reactive astrogliosis can be seen in Figure 1). Some researchers reported that H_2_O_2_, a potent oxidant, can upregulate the expression of GFAP and ROS in astrocytes in vitro, and the intervention of molecular hydrogen could inhibit both the production of ROS and overexpression of GFAP in astrocytes.^67^ Rotenone is a direct mitochondrial respiratory chain blocker, and as Goswami et al^37^ found, it could upregulate the expression of GFAP in C6 astrocytoma cell line by increasing the mitochondrial ROS. Some inflammation inducers like LPS can stimulate astrocytes to produce NO (a main member of RNS) and increase GFAP expression.^12^ In an in vivo study, a cortical transplantation model was used to mimic glial activation in neurodegenerative diseases and the results suggest that sustained high levels of oxidative stress after transplantation are associated with chronic glial activation.^7^ MitoQ, a mitochondria‐targeted antioxidant, attenuates excessive reactive astrogliosis in the brains of Alzheimer's disease model of mice.^78^ Additionally, some amino acid complex, like cysteamine (CSH), promotes mitochondrial oxidative stress in astrocytes and causes astrocyte activation both in vivo and ex vivo, and the authors indicated that intervention of CSH can be used as a model to study reactive astrogliosis induced by ageing and neurodegenerative diseases.^74^ JAK‐STAT signal pathway is a key direct mediator of astrogliosis.^91^, ^98^, ^101^ Conditional knockout STAT3 in astrocytes leads to inactivation of astrocytes, and there is no glial scar formed in the injury site of astrocytic STAT3 conditional knockout mice in spinal cord injury model.^137^ The activation of JAK‐STAT signal was reported to be affected by oxidative stress.^137^ In a Parkinson's disease model, enhanced NOX activity in microglia is able to mediate microglia‐induced reactive astrogliosis by activating STAT1 or STAT3 signalling pathways.^45^ On the contrary, activation of endogenous antioxidant system like Nrf2‐Keap1‐ARE could inhibit the activation of excessive astrogliosis.^47^, ^141^ In conclusion, regulation of oxidative stress may be an effective way to inhibit excessive glial cell activation and glial scar formation.

### 5.3 Effect on glutamate transport

As reviewed in the previous section, glutamate metabolism in astrocytes plays a crucial role in maintaining the balance of oxidative stress in the CNS. Reciprocally, the oxidative stress level in CNS also significantly affects the metabolism of glutamate by astrocytes. Studies have found that in vitro peroxide intervention is able to reduce both the glutamate transporter of astrocytes and the transport capacity of astrocytes to glutamate.^81^ Antioxidants such as hydrogen sulphide and propofol can alleviate the inhibition effect of glutamate transporters induced by oxidative stress and thus maintain the transport of glutamate to astrocytes.^72^, ^116^ Korcok J found that LPS and IFN γ treatment significantly inhibit the astrocytic glutamate uptake and this effect could be reversed by ascorbate, an endogenic antioxidant.^61^ Jayakumar et al^51^ found that astrocytic oedema and decreased glutamate uptake of astrocyte are related to the activation of oxidative stress‐dependent MAPK pathway, while the antioxidase and antioxidants such as SOD, catalase and vitamin E can inhibit MAPK activation and significantly reduce cell oedema and enhance glutamate uptake. Another study indicated that amyloid‐β protein (Aβ) could reduce the glutamate uptake of astrocytes in vitro by increasing oxidative stress and MAPK activation.^75^ Reactive astrogliosis and dysfunctional transporters for l‐glutamate [excitatory amino acid transporters, (EAATs)] are the hallmarks of amyotrophic lateral sclerosis (ALS) pathology. Zagami et al^145^ found that oxidative stress leads to early astrogliosis and impaired EAAT activity of astrocyte, pointing to a critical role of astrocytes in response to oxidative‐induced injury in an ALS model.^145^ In addition to its effects on glutamate uptake, ROS also affects astrocytic glutamate secretion and this might be one of the potential reasons leading to the neurotoxicity of hippocampal neurons induced by ethanol.^108^ Except for the influence of ROS, RNS is proved to be associated with glutamate metabolism.^97^ The results of some studies confirm that the both glutamate transport and metabolism in glutamate/glutamine cycle in astrocytes can be regulated by NO and NO‐induced cysteine S‐nitrosylation.^97^, ^142^

## 6 ROLE OF ASTROCYTE‐RELATED OXIDATIVE STRESS IN CENTRAL NERVOUS SYSTEM DISEASES

### 6.1 Traumatic injury of central nervous system

Traumatic injury of CNS is always accompanied with severe inflammation and oxidative stress, which lead to the so‐called "secondary strike" effect.^50^, ^83^, ^131^ The drastic change of redox level after injury significantly affects the physiological function of astrocytes. For example, lower astrocytic glutamate transporter (EAATs) expression has been found in human brain biopsy tissue among traumatic brain injury patients, suggesting that the ability of astrocytic excitatory amino acid uptake might be decreased after injury.^58^ As reviewed in the former section, accumulation of excitatory amino acid in the microenvironment may lead to the overload of mitochondrial calcium, which aggravates the oxidative stress and neuron damage. Lu et al confirmed that the reducing substances such as H_2_S could increase the expression of glutamate transporter (GLT‐1) and reduce the production of ROS, thereby reducing secondary damage after mechanic injury.^134^ Ahmed et al^3^ found that traumatic tensile stress could cause mitochondrial dysfunction in astrocytes and the damaged astrocytes can further affect neuronal mitochondrial function. Additionally, astrocytic NO production significantly increased after injury and it is closely related to the secondary injury.^79^ Inhibition of NOS activity is reported to be an effective way to reduce neural damage.^95^ The antioxidant system like Nrf2 system is reported to be significantly activated, alleviating oxidative and inflammatory damage in traumatic brain injury and spinal cord injury.^92^, ^136^ Therefore, targeting astrocytes might be an effective way to reduce excessive oxidative stress and further alleviate secondary injury induced by traumatic CNS injury.

### 6.2 Stroke

Stroke is a clinical condition that results in CNS cell death because of the poor blood flow to the CNS. According to the aetiology, stroke can be divided into haemorrhagic stroke and ischaemic stroke. Stroke is the leading disabling and fatal diseases worldwide, and oxidative stress is the main factor leads to cell damage induced by stroke.^59^ Astrocytes are reported to play both neuroprotective and destructive roles in the whole pathological process of stroke by altering their cell structure and physiological function in response to the change of microenvironment induced by stroke. As the important homeostatic cells in the CNS, astrocytes are widely involved in oxidative stress modulation after stroke. On the one hand, the antioxidant response of astrocytes may save neurons from excessive oxidative stress after stroke: astrocyte‐specific overexpression of superoxide dismutase 2 (SOD2) effectively attenuates nerve damage caused by cerebral ischaemia.^140^ Hayakawa et al^42^ found that astrocytes can deliver their own functional mitochondria to neurons directly or indirectly, and blocking this process might aggravate neural damage arising from cerebral ischaemia. This phenomenon reveals a new significant mechanism for the direct regulation of oxidative stress in glial cells, and it may provide a new target for the improvement of endogenous neuroprotection after stroke. On the other hand, excessive oxidative stress induced by stroke in the central nervous system after stroke can in turn cause activation of astrocytes, which might finally form glial scars and impede neurological recovery.^20^, ^65^ In addition, activated astrocytes secrete a variety of pro‐inflammatory factors aggravating the secondary inflammatory response after stroke.^106^ Gouix et al found that oxygen glucose deprivation leads to dysfunction of glutamate uptake in astrocytes in vitro, which aggravate excessive oxidative stress to result in cell death. To sum up, astrocytes act as different roles in different stages of the stroke pathology and oxidative stress might be one of the important factors resulting in this difference.

### 6.3 Neurodegenerative diseases

Neurodegenerative disease is a series of CNS diseases arising from progressive loss of neurons and/or myelin, deteriorating over time and causing neural dysfunction, with an increasing high mobility over the world and including a wide range of CNS disease such as Alzheimer's disease (AD), Parkinson's disease (PD), amyotrophic lateral sclerosis (ALS), Huntington's disease (HD). Astroglial asthenia often leads to the disturbance of CNS homeostasis which is reported to be highly related to these diseases.^129^

Alzheimer's disease is the most common neurodegenerative disorder in the world and a leading cause of dementia. Oxidative stress and astrocytes are both deeply involved in the development of the pathology of AD. The extracellular deposition of neuritic plaques and intracellular accumulation of neurofibrillary tangles which, respectively, formed by β‐amyloid and abnormal tau phosphorylation are considered as the primary neuro‐pathogenesis of AD.^71^ Oxidative stress has been shown in a wide range of studies to be participant in these pathogenesis of AD.^14^ Some in vitro studies indicated that the accumulation of Aβ could increase the production of ROS in astrocytes and exacerbate neural damage.^56^, ^111^ Regulation of astrocytic mitochondrial function by cytokines like insulin‐like growth factor‐1 (IGF‐1) is associated with learning and memory.^70^ Except for ROS, NO is proved to be released around the Aβ plaques by astrocytes.^132^ Lipoproteins from Alzheimer patients can lead to the increased production of peroxynitrite and NOS in astrocytes.^88^

Parkinson's disease is the second most common neurodegenerative disease over the world.^102^ As for its pathogenesis, α‐synuclein is considered as one of the most important pathogenesis‐related proteins and the researchers found that α‐synuclein could be transferred from neurons to astrocytes in PD model.^63^, ^119^, ^128^ Direct injection of human α‐synuclein into the basal ganglia of mice could lead to strong astrocytes and microglia activation.^124^ An in vitro study shows that alpha‐synuclein could induce generation of abundant ROS and inflammatory factors mediated by activation of TLR4 receptor of astrocyte.^29^ Besides, accumulation of α‐synuclein could aggravate oxidative stress in a of astrocyte‐neuron co‐culture systems, causing lipid peroxidation and death of neurons.^6^ Aberrant S‐nitrosylation is also reported to be associated with PD pathogenesis: S‐nitrosylated parkin, a PD associated protein, can be found in the brain of PD patient and mouse models of PD^21^; however, the potential role of astrocytes in this pathology remains unclear.^102^

As for ALS study, approximately 90% of familial ALS patients have mutations in SOD1 which encode an important antioxidant enzyme.^64^ An experimental ALS study shows that astrocytes expressing mutant superoxide dismutase 1 are toxic to normal motoneurons.^31^ In addition, SOD1 mutant astrocytes could secrete more TGFβ, expressing higher level of inflammasomes like NLRP3, and activate NF‐KB signalling pathway to aggravate the inflammatory response in an ALS model.^64^ Compared to the control group, astrocytes expressing mutant SOD1 and TDP43 result in worse nitroxidative stress which cause more death of motoneurons.^103^ In conclusion, astrocytes are one of the potential targets for oxidative stress regulation to improve the outcome of neurodegenerative diseases.

## 7 SUMMARY

In summary, the health state of CNS is closely associated with the balance between oxidative and antioxidative factors. Astrocytes as the main supportive cells in CNS are significantly involved in the redox homeostasis maintaining under physiological or pathological conditions. Accumulating evidence shows that astrocytes play a dual role in ROS/RNS regulation: on the one hand, astrocytes could protect the central nervous system from oxidative injury by producing various antioxidant, removing the excitatory amino acids and activating some endogenic antioxidative systems like Nrf2 as the neuroprotective role. On the other hand, under certain circumstances, astrocytes could be an important source of excessive ROS and RNS because of mitochondrial dysfunction, impaired excitatory amino acid metabolism and antioxidant generation, which plays a detrimental role. The pathogenesis of numerous neurological disorders, such as stroke, trauma, infection and neurodegenerative diseases, is reported to be highly associated with astrocytic redox homeostasis. Reciprocally, excessive free radicals in the microenvironment of CNS may lead to reactive astrogliosis aggravating inflammation and glial scar formation, both of which burden the CNS. Increasing evidence indicates that astrocyte might be a promising target for oxidative stress modulation in CNS and this may provide us with future therapies on those related diseases. Additionally, personalized intervention needs to be considered because of the different effects of astrocytes under different conditions. Understanding the mechanisms of CNS redox biology and the relative role of specific cell types will pave the way for effective therapeutics targeting for oxidative stress in CNS disorders.

## CONFLICT OF INTEREST

The authors have no competing interests to disclose.

## AUTHOR CONTRIBUTIONS

LZ, AT, JX and GG contributed to conception and design of the study; YC and CQ wrote the first draft of the manuscript; JH and XT wrote sections of the manuscript; and CL and KH searched the literature. All authors revised the manuscript and approved the submitted version.

## Data Availability

Research data are not shared.
